# Supplementary figures and images for: Respiratory Bacteria Stabilize and Promote Airborne Transmission of Influenza A Virus
Source: mSystems. 2020 Sep 1;5(5):e00762-20. doi: 10.1128/mSystems.00762-20 (PMC7470989; doi:10.1128/mSystems.00762-20)

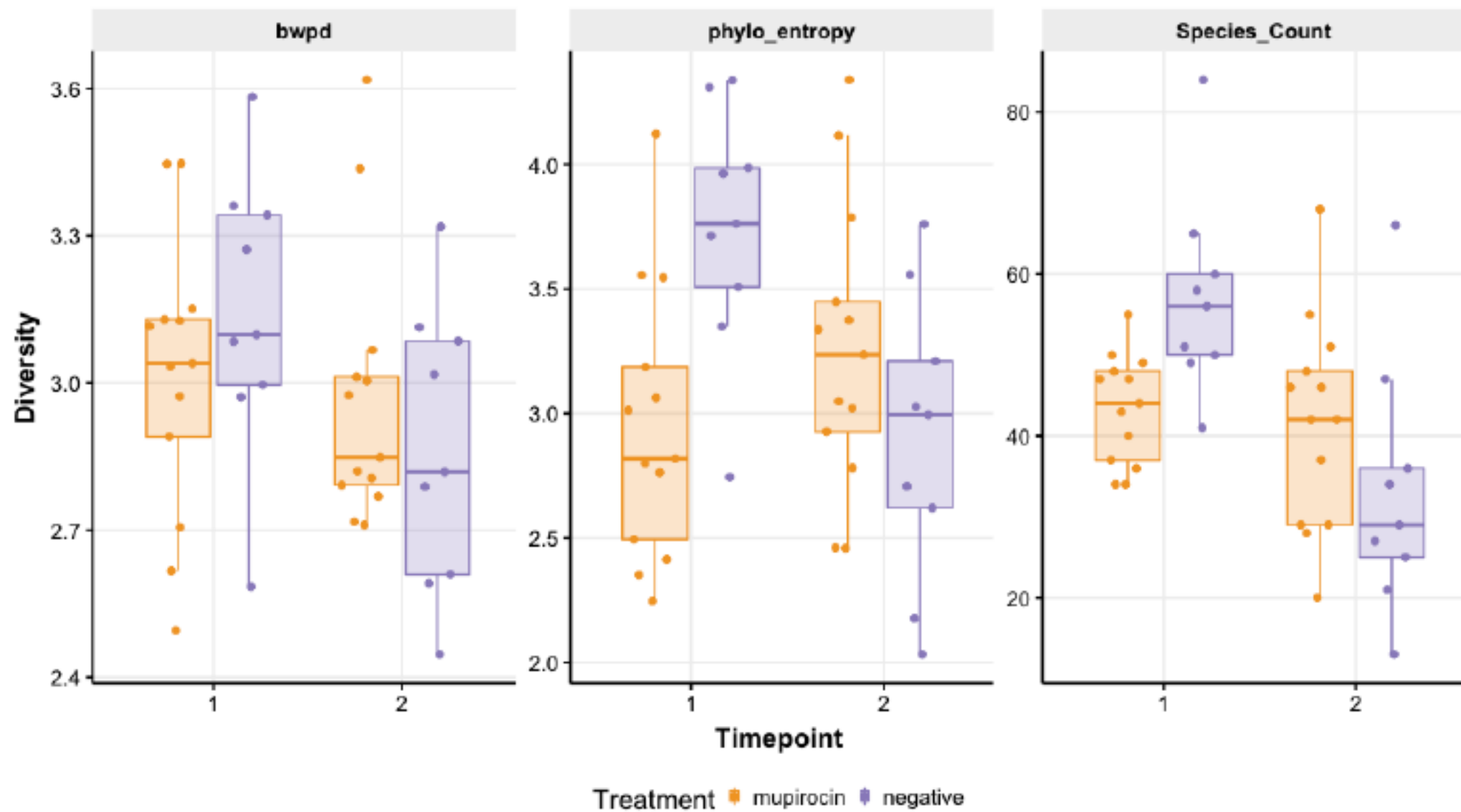

Supplement: FIG S1 [file mSystems.00762-20-sf001.pdf]

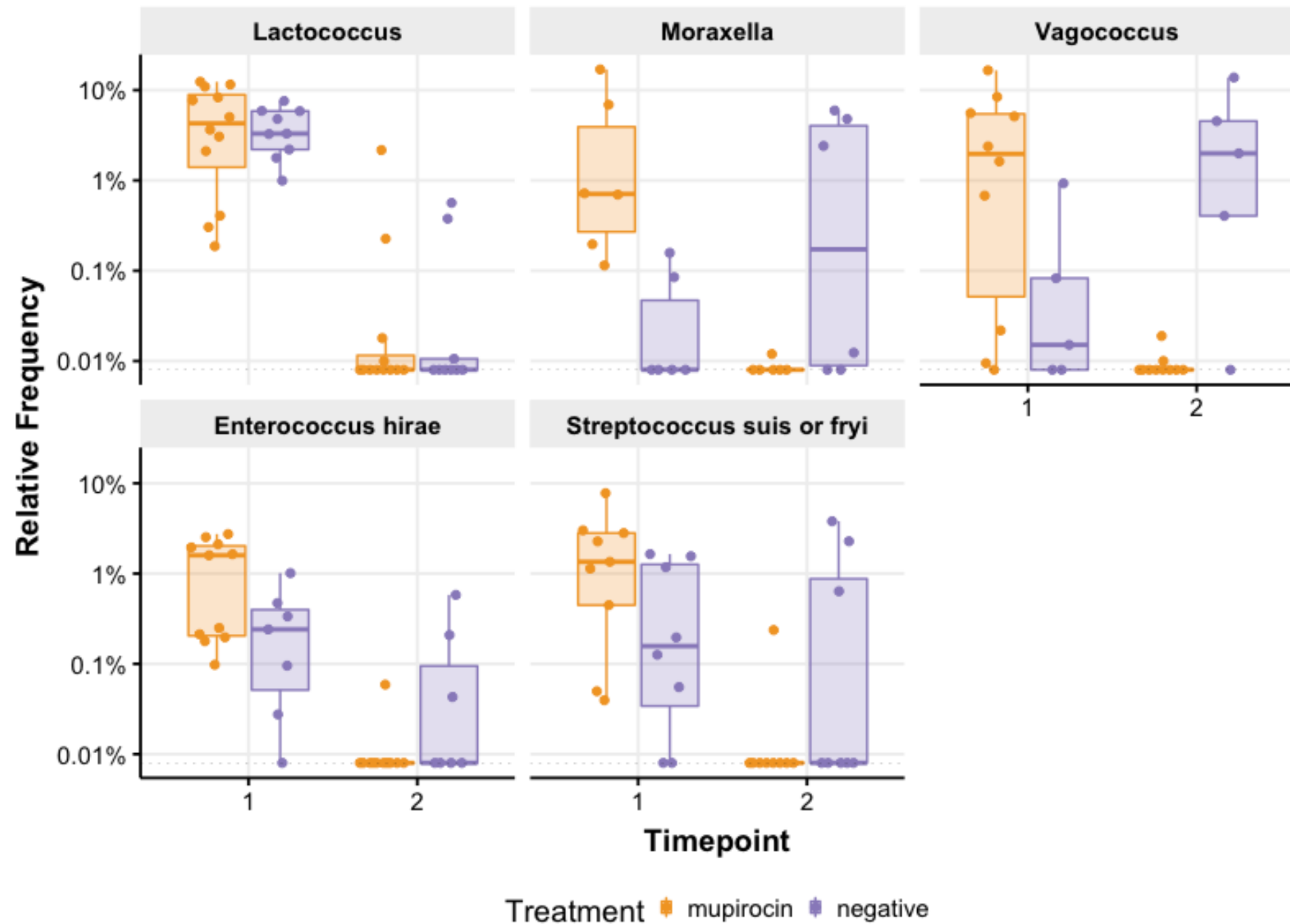

Supplement: FIG S2 [file mSystems.00762-20-sf002.pdf]

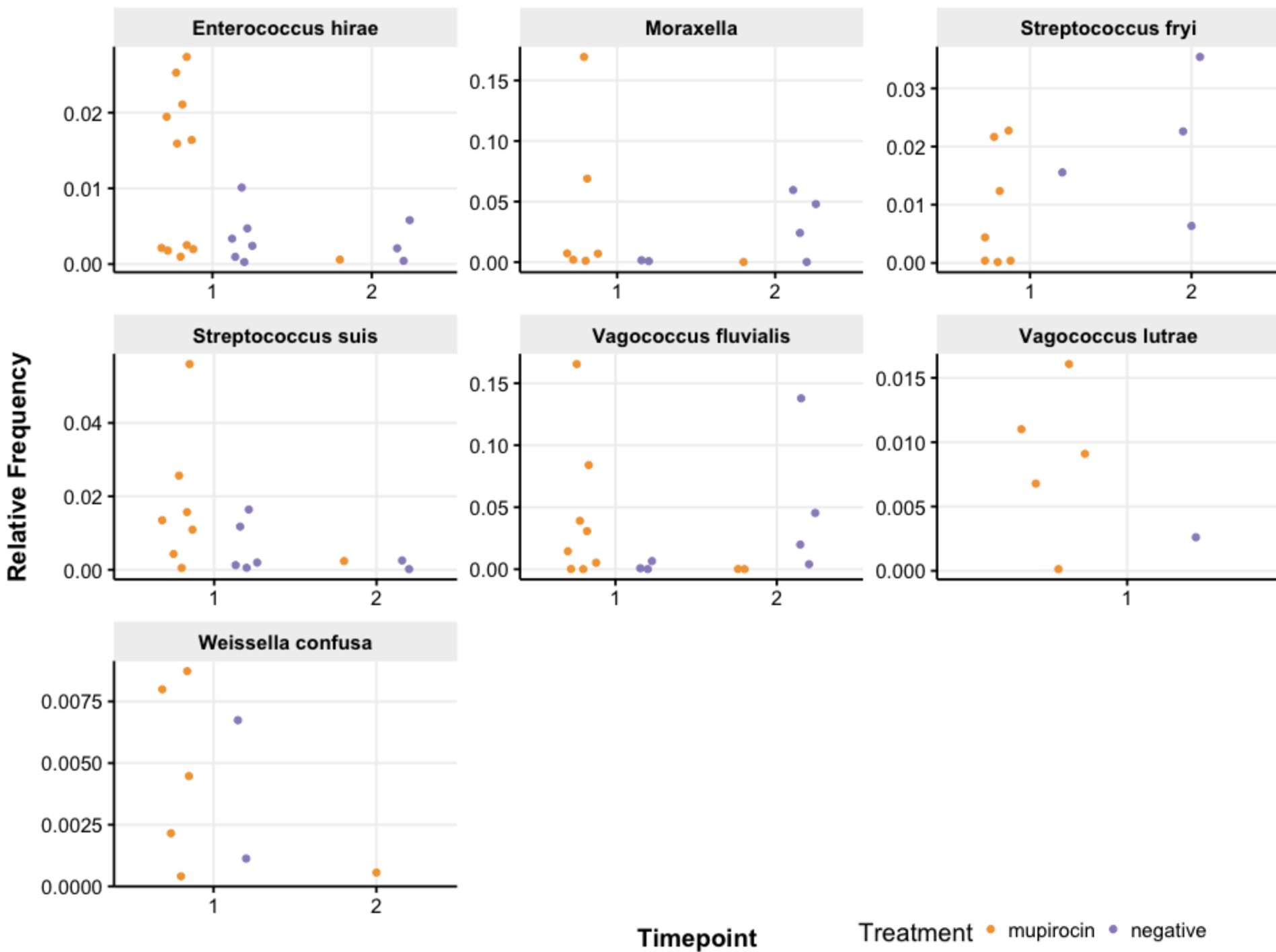

Supplement: FIG S3 [file mSystems.00762-20-sf003.pdf]

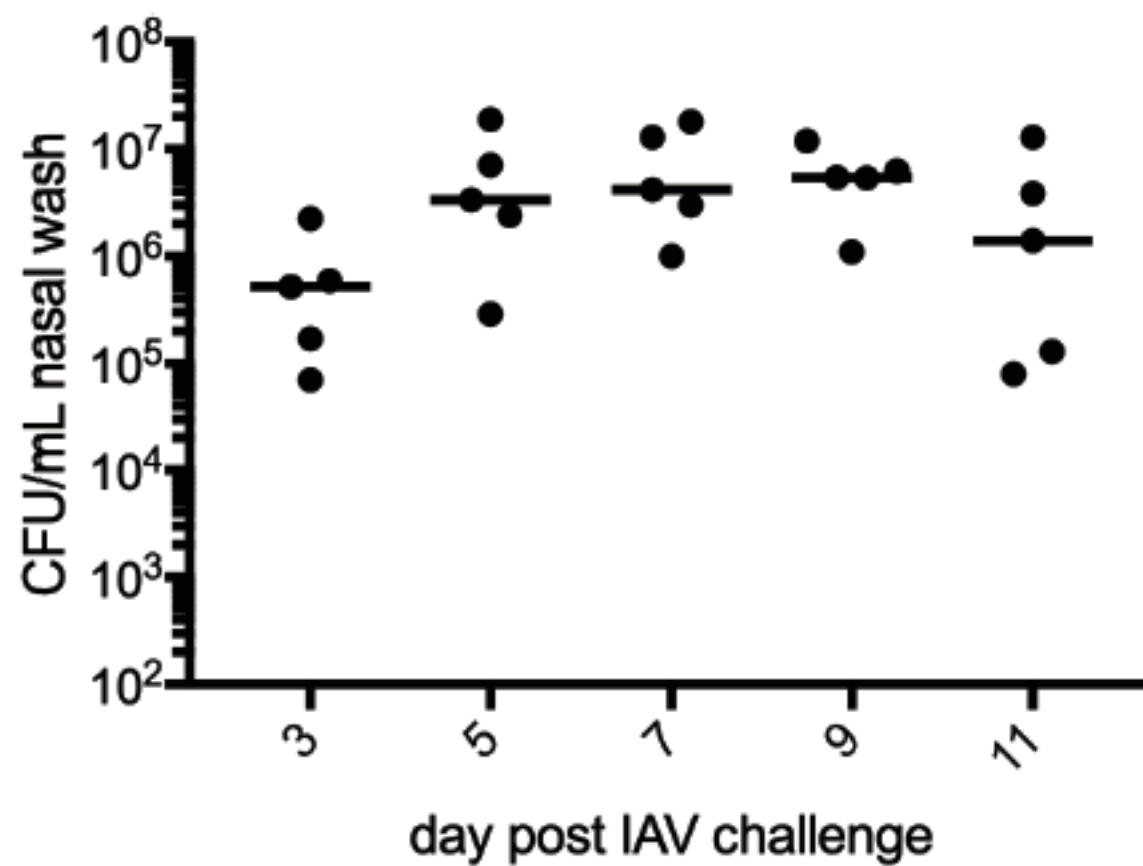

Supplement: FIG S4 [file mSystems.00762-20-sf004.pdf]
