# Supplementary material for: Respiratory Bacteria Stabilize and Promote Airborne Transmission of Influenza A Virus
Source: mSystems. 2020 Sep 1;5(5):e00762-20. doi: 10.1128/mSystems.00762-20 (PMC7470989; doi:10.1128/mSystems.00762-20)
Supplement: TABLE S1 [file mSystems.00762-20-st001.pdf]

Supplemental Table 1: Symptom scores of donor ferrets

Ferret ID	Max Weight loss (% starting weight)	Max temperature, fever[a] bold text	Max lethargy score[b]	Days with Sneezing	Days with nasal/ocular discharge
Untreated 1	5.8	38.9	0	7	3
Untreated 2	6.1	**39.6**	0	1	0
Untreated 3	6.5	39.2	0	1	1
Untreated 4	3.4	38.9	0	3	0
Untreated 5	0.3	39.3	0	3	0
Untreated 6	1.4	39.3	0	3	0
Untreated 7	11.6	38.7	0	4	0
Mupirocin treated 1	6.5	38.6	1	8	1
Mupirocin treated 2	12.0	**39.6**	0.5	7	0
Mupirocin treated 3	6.2	**39.6**	0	5	0
Mupirocin treated 4	2.2	38.4	0	4	0
Mupirocin treated 5	1.6	38.7	0	3	1
Mupirocin treated 6	0.9	**39.7**	0	4	1
Mupirocin treated 7	7.6	38.5	0	4	0
Mupirocin treated 8	0.4	39.0	0	4	0
Vehicle treated 1	0.6	38.6	0	2	0
Vehicle treated 2	4.9	38.9	0	7	0
Vehicle treated 3	8.7	39.3	0	4	0
S.pneumoniae infected 1	7	39.2	1	7	4
S.pneumoniae infected 2	2.6	39.4	1	6	4
S.pneumoniae infected 3	8.0	**40.3**	1	9	4
S.pneumoniae infected 4	23.3	**39.8**	0	9	5
S.pneumoniae infected 5	20.1	39.4	0	7	5

[a] Fever defined by temperature greater than 39.5°C

[b] Lethargy score: 0= alert and playful, 1=alert, playful when stimulated, 2=alert, not playful when stimulated, 3=neither alert nor playful when stimulated
